# The Challenges and Opportunities for Performance Enhancement in Resonant Fiber Optic Gyroscopes

**DOI:** 10.3390/s25010223

**Published:** 2025-01-03

**Authors:** Sumathi Mahudapathi, Sumukh Nandan R, Gowrishankar R, Balaji Srinivasan

**Affiliations:** 1 Avionics Division, Department of AFCS, Hindustan Aeronautics Ltd., Hyderabad 500042, India; ee17d054@smail.iitm.ac.in; 2 Department of Electrical Engineering, Indian Institute of Technology Madras, Chennai 600036, India; 3 Department of Physics, Sri Sathya Sai Institute of Higher Learning, Sri Sathya Sai District 515134, India; sumukhnandanr@sssihl.edu.in (S.N.R.); rgowrishankar@sssihl.edu.in (G.R.)

**Keywords:** inertial navigation, resonant fiber optic gyroscope, bias stability, frequency comb source, single coupler resonator with reflector, hollow core anti-resonant fiber

## Abstract

In the last decade, substantial progress has been made to improve the performance of optical gyroscopes for inertial navigation applications in terms of critical parameters such as bias stability, scale factor stability, and angular random walk (ARW). Specifically, resonant fiber optic gyroscopes (RFOGs) have emerged as a viable alternative to widely popular interferometric fiber optic gyroscopes (IFOGs). In a conventional RFOG, a single-wavelength laser source is used to generate counter-propagating waves in a ring resonator, for which the phase difference is measured in terms of the resonant frequency shift to obtain the rotation rate. However, the primary limitation of RFOG performance is the bias drift, which can be attributed to nonreciprocal effects such as Rayleigh backscattering, back-reflections, polarization instabilities, Kerr nonlinearity, and environmental fluctuations. In this paper, we review the challenges and opportunities of achieving performance enhancement in RFOGs.

## 1. Motivation

Gyroscopes are at the core of inertial navigation, guidance, and control in a wide variety of vehicles such as aircraft, ships, submarines, and automobiles. The past 30 years of research around the world have resulted in the development of many types of gyroscopes, including mechanical, optical, and MEMS-based technologies [1,2,3,4]. However, there is a trade-off between cost and performance in terms of the bias stability, scale factor stability, and angular random walk (ARW) of gyroscopes for navigation, control, and guidance applications. For instance, strategic missile navigation and submarine navigation applications require inertial grade gyroscopes with a scale factor stability of the order of less than 50 ppm, as well as a bias stability of less than 0.01°/h. On the other hand, for autonomous navigation in low-budget industrial applications, a tactical-grade gyroscope with a bias stability of 10°/h is sufficient [5,6]. The performance requirements for various grades of gyroscopes are mentioned in Table 1.

**Figure 1:**
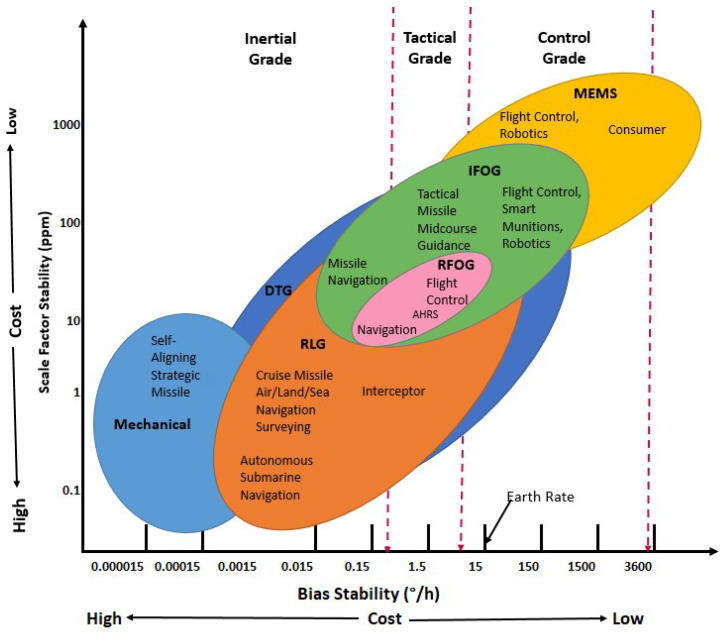
Performance comparison of various gyroscope technologies used for inertial/tactical/ control grades; adopted from [4].

An overview of the various applications of gyroscopes and their corresponding performance requirements is presented in Figure 1 [4]. As can be seen from Figure 1, gyroscopes with different working principles and technologies are classified based on their performance, with an associated increase in cost. The top-right corner of the plot is occupied by MEMS-based gyroscopes, which have been successfully established in the market over the past decade for control-grade applications at large-scale production [7,8]. However, their long-term bias stability issues and their low sensitivity have prohibited their use in high-performance applications. On the other hand, mechanical gyroscopes provide excellent bias stability and scale factor stability and are widely used for inertial-grade applications. However, they are bulky and have movable parts that drift over a period of time due to ‘g’ sensitivity. Optical gyroscopes pose an excellent alternative to their mechanical counterparts since they are much more compact and are relatively less prone to drift issues. Of the different optical gyroscopes listed in Figure 1, ring laser gyroscopes (RLGs) provide the best performance, with a bias stability closer to that of mechanical gyroscopes, and they are widely deployed in inertial grade applications. However, for tactical-grade applications, interferometric fiber optic gyroscopes (IFOGs) are preferred as they are much more amenable to mass manufacturing and have recently demonstrated a bias stability value closer to that of RLGs used for navigation applications [9].

The performance characteristics of IFOGs are largely dependent on the optical components chosen and the fiber coiling techniques used. Various research groups worldwide have demonstrated novel designs with improvements made to existing fiber optic gyroscope (FOG) technology [10,11]. Research over the past decade has highlighted the fact that FOGs made of resonators instead of interferometers could be a cost-effective alternative in the FOG market [2,12]. Novel ways of exploiting the Sagnac effect have also been reported [13,14]. Such work opens up new avenues for drastically reducing the sensing fiber length (kilometer-scale in IFOGs), along with novel designs, to overcome nonreciprocal errors without compromising sensitivity. Although IFOGs are the most successfully deployed version among FOGs, several new configurations, including RFOGs and resonant micro-optic gyroscopes (RMOGs), are being explored to enhance the performance of the gyroscope [15]. Specifically, research into RFOGs has recently gained momentum [16,17,18,19], with the aim of matching IFOG and mechanical gyro performance, especially in applications where there is demand for smaller sizes. Since RFOGs use shorter lengths of fiber compared to IFOGs [20], the drift due to time-variant temperature distribution (referred to as the Shupe effect) is reduced, thus also reducing the sensing coil cost. One of the primary factors impacting the bias stability of a conventional RFOG based on a high coherence source is laser phase noise [21,22,23]. Recent developments in RFOGs have focused on improving bias stability to match that of mechanical gyroscopes. In this review article, we discuss the fundamental working principle of RFOGs and their typical configurations, followed by a discussion on the key challenges that limit the performance of RFOGs. Finally, we present opportunities for performance enhancement in RFOGs.

## 2. Working Principle of Resonant Fiber Optic Gyroscopes

**Figure 2:**
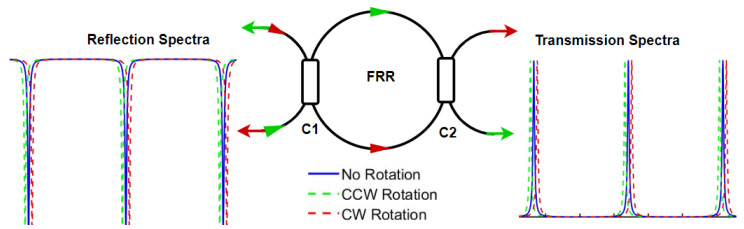
A schematic diagram illustrating the working principle of resonant fiber optic gyroscopes (RFOGs). Any rotation experienced by the fiber ring resonator (FRR) will result in an upward or downward shift in the resonance frequency for the two counter-propagating waves (CW and CCW, respectively). This frequency shift may be demodulated into an intensity change by typically locking the source frequency with respect to the FRR resonance.

The resonant fiber optic gyroscope (RFOG), which was first studied by Ezekiel et al. in 1977 is a passive resonator-based rotation sensor interrogated with a light source placed externally to the cavity [24]. The spectral response of a ring resonator, as presented in Figure 2, shows longitudinal modes or resonances separated by a uniform frequency spacing known as free spectral range (FSR), which may be observed through the light coupled out of the two couplers (C1 and C2) in the reflection mode and transmission mode, respectively. The optical resonance takes place when the counter propagating light beams are phase shifted by an even multiple of π. The expression for cavity resonance frequency is given by $\nu_{q}$ = $qc/(\pi Dn_{eff})$, where *q* is the resonance order, *c* is the velocity of light in free space, *D* is the diameter of the ring resonator, and $n_{eff}$ is the effective refractive index seen by the counter-propagating waves. When the resonator is at rest, the resonance frequency is the same for the clockwise (CW) and counter clockwise (CCW) propagating light beams. However, when the resonator is subjected to rotation, the resonance frequency of the two counter propagating light beams changes in the opposite direction, resulting in a frequency difference, Δν, between them. Thus, by measuring the resonance frequency difference, Δν, we can infer the rotation rate Ω that the resonator is experiencing. The frequency difference between the two counter-propagating light beams expressed in Equation (1) is independent of the number of turns and only depends on the diameter of the cavity.

(1) $$\Delta \nu =\nu_{cw}-\nu_{ccw}=\frac{D}{n_{eff}\lambda }\Omega$$

A key requirement of conventional RFOGs is the locking of the source laser frequency with respect to the resonance of the FRR such that the frequency change due to rotation may be demodulated as an optical intensity change. This may be achieved by independently modulating the CW and the CCW propagating light beams—either phase modulation [25,26,27] or frequency modulation [28]—and performing lock-in detection to improve the signal-to-noise ratio.

**Figure 3:**
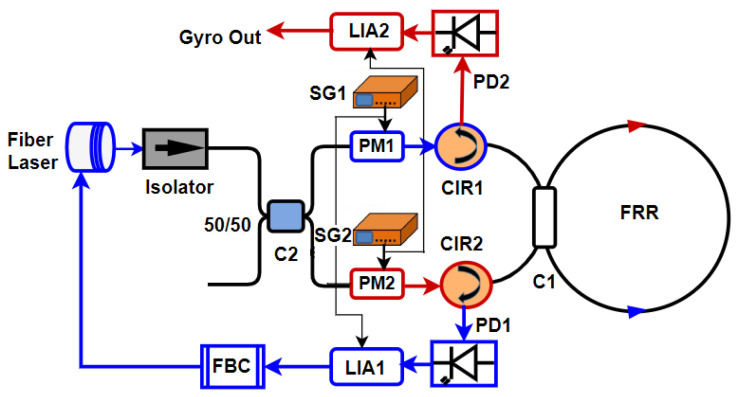
A typical lock-in detection-based readout system for RFOGs (reproduced from [26]). The input CW and CCW light beams are either phase or frequency-modulated to enable lock-in detection. One of the output signals (CCW in the above figure) is used to keep the source laser locked to the FRR resonance, whereas the other (CW) is used to read out the rotation rate.

A typical RFOG readout system is shown in Figure 3. The source laser beam is split into two using an optical splitter/coupler and an isolator. The two light beams are generally phase or frequency modulated using two separate modulators (PM1 and PM2) such that the modulated light spectra are distinct and do not overlap. The two light beams are then launched from the two opposite directions (CW and CCW) of the resonator. The two output light beams from the FRR, for which the intensity is modulated according to the rotation rate, are captured using separate photodetectors (PD1 and PD2) and fed into lock-in amplifiers to extract the baseband signals corresponding to the rotation rate. One of those signals (CCW in Figure 3), which is extracted using the circulator CIR2, is used to fine tune the source laser frequency with respect to the FRR resonance frequency, whereas the other output (CW) extracted using circulator CIR1 is read out as the rotation rate of the RFOG. One of the key parameters that quantifies RFOG performance is the minimum detectable rotation rate or RFOG sensitivity. Similar to IFOGs, the shot noise-limited rotation rate sensitivity of an RFOG is given by

(2) $$\delta \Omega =\delta \nu \frac{\lambda }{D}\sqrt{\frac{2Bhc}{\eta \lambda P_{pd}}}$$

where *B* is the detection bandwidth of the gyroscope, *D* is the fiber loop diameter, and $P_{pd}$ is the power received at the photodetector. It is interesting to note from Equation (2) that the minimum detectable rotation rate is decided by the full-width half maximum (FWHM) of the cavity, δν. For example, to realize a sensitivity of 0.1°/h, an RFOG typically requires a laser with a linewidth of <10 kHz. In other words, the angular rotation rate measurement in the above RFOG configuration requires a laser linewidth much smaller than the spectral width of the cavity resonance. Recent developments in narrow linewidth lasers have focused on reducing the laser phase noise while maintaining high output power [29]. This is typically achieved by introducing a micro-resonator as part of the laser cavity.

## 3. Key Implementation Challenges for Resonant Fiber Optic Gyroscopes

For high-performance inertial-grade applications, a gyroscope requires the bias stability, scale factor stability, and ARW values to be as low as possible. During the past 30 years, different research groups around the world have focused on determining the key factors limiting the above performance parameters in RFOGs and have identified the following: Rayleigh backscattering, polarization fluctuations in the ring resonator, the optical Kerr effect, the stabilization of the source laser frequency, and the post-processing of the acquired data. In this section, we review the challenges posed by each of the above factors and discuss the solutions implemented to mitigate them and enhance RFOG performance.

### 3.1. Rayleigh Backscattering

Rayleigh backscattering refers to the scattering of light in a backward direction due to microscopic inhomogeneities in fused silica fiber. Such backscattering gives rise to a transfer of power between the CW and CCW propagating beams [30]. As such, the Rayleigh backscattered light spectrum exhibits two peaks when the FRR is subjected to rotation—one of the two peaks corresponds to the shift in resonance due to rotation, and the other peak corresponds to the original resonance frequency due to the Rayleigh backscattering. The backscatter coefficient decides the extent of fluctuation in the gyroscope output. For instance, in a fiber coil of 1 km, the backscattering can lead to a power fluctuation of 40 dB [31]. In the case of RFOGs, certain carrier suppression techniques are implemented to minimize the backscattering effects [32]. Such backscattered radiation also degrades RFOG linearity, as it produces enhanced noise at different input rotation rates. The strength of the backscatter coefficient decides the extent of fluctuation in the gyroscope output. This noise is eliminated by phase or frequency modulating the laser source using different modulation frequencies for the CW and CCW-propagating beams [33,34]. In previous work, an 80 dB carrier suppression ratio has been demonstrated for a reduction in backscattering induced noise in RFOGs, which corresponds to a phase bias drift of as low as $10^{-6}$ rad/s [35]. In other work, the triangle phase modulation method [36,37] and double phase modulation method [38] have been demonstrated to reduce the Fresnel backreflections at the splicing point of a ring resonator with a coupler. A side-band locking method has also been implemented [39] to reduce backscattering in RFOGs, along with bias compensation methods [40].

### 3.2. Polarization Fluctuations

Polarization instabilities lead to nonreciprocal errors in RFOGs. Theoretically, a single-mode optical fiber used in RFOGs has two different Eigen states of polarization, which lead to two distinct effective indices and resonant frequencies. Though all the optical power is launched at one particular polarization, the orthogonal state of polarization also gets excited due to thermal effects and bends in the fiber loop. The unwanted polarization moves towards the resonance of the primary polarization state and, over a long time, causes drift, which becomes indistinguishable from the rotation induced frequency shift [41,42]. This effect creates long term instability in RFOGs and can be reduced by using polarization-maintaining fiber (PMF), decreasing polarization crosstalk in the resonator, better alignment of any splice in the resonator, and decreasing the difference between the coupling and loss coefficients of the resonator output coupler [1,42,43,44,45,46,47].

### 3.3. Optical Kerr Effect

The optical Kerr effect or Kerr nonlinearity refers to the variation in the refractive index optical intensity at any particular location in the sensing fiber. The optical Kerr effect is more prominent in RFOGs compared to IFOGs due to the high finesse of the resonator and the resulting resonant enhancement of the optical intensity. For this reason, Kerr nonlinearity poses a major limitation to RFOG sensitivity [48,49]. A square wave modulation technique has been attempted to reduce the Kerr effect, but the implementation of this is tricky due to the need for precise modulation [50]. Hollow core photonic bandgap fibers are good candidates for reducing the effect of Kerr nonlinearities in RFOGs [51,52,53], but they are expensive and pose a challenge in terms of integration with other conventional fiber based components. The equalization of the counter propagating beam intensities by servo control and relative intensity noise compensation can potentially reduce the Kerr effect [50,54].

### 3.4. Laser Frequency Stabilization

In a conventional RFOG configuration, the center frequency of the laser source is tuned to the resonance frequency of the ring resonator. The source frequency fluctuations cause resonance frequency fluctuations and induce the bias drift. By using an active frequency stabilization approach, such as the Pound-Drever-Hall (PDH) technique, the source center frequency is kept equal to the resonant frequency of the ring resonator [55]. In RFOGs, the Shupe effect is not a major concern due to the shorter lengths of fibers involved [56,57]. However, the bias drift due to temperature is a limiting factor, and it can be eliminated by using a servo loop with a PI controller [58]. Geng et al. demonstrated the all-optical frequency locking of two DFB lasers to the CW and CCW resonances using a self-injection method; they measured the beat frequency using a time-to-digital converter chip to improve bias stability [59].

### 3.5. Signal Processing

**Figure 4:**
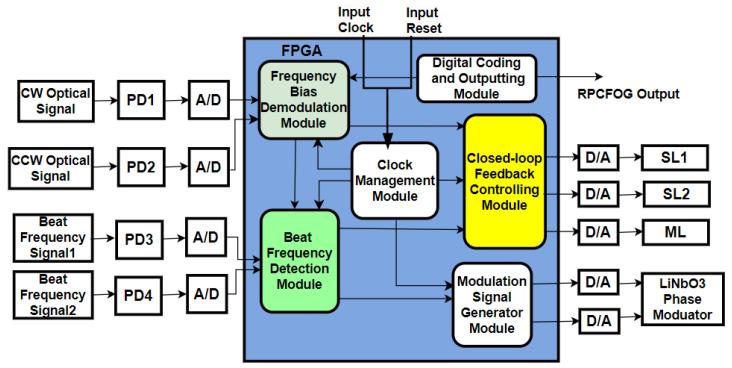
Schematic diagram illustrating the various algorithmic blocks implemented in an FPGA for signal detection [20].

Various signal-processing methods have been implemented to detect rotation rate, including cavity length modulation, frequency modulation, phase modulation spectroscopy, lock-in amplifiers, and digital modulation demodulation implementation in FPGA [20]. A typical signal-detection logic circuit is shown in Figure 4. The algorithm corresponding to servo control logic, signal generation for phase modulation, and beat signal detection logic are implemented in an FPGA to improve the signal-to-noise ratio and thereby improve rotation rate measurement accuracy. In another study, a particle swarm optimization algorithm was used to optimize the multiple parameters involved in rotation rate measurement, resulting in ARW improvement [37].

### 3.6. RFOG Performance Evolution

The research and development efforts related to RFOGs over the past decade have addressed most of the above challenges, and the focus is on improving the following parameters: (i) Sensitivity: the minimum rotation rate that can be measured. The shot noise-limited rotation rate is the minimum value that can be measured, typically. (ii) Bias Stability: when the system is at rest, the gyroscope measures a value referred to as the bias in the system. This bias value changes with noise and degrades RFOG performance over a time period. The bias stability is typically quantified with the help of an Allan deviation curve. (iii) Angular random walk (ARW): the white noise present in the system increases the error in measuring the rotation rate over a time period, which is known as the angular random walk.

The details of various research efforts carried out to date to improve the sensitivity of RFOGs are presented in Table 2. Meyer R et al., 1983, demonstrated an RFOG using a He-Ne laser and a single-mode fiber-based ring resonator and achieved a sensitivity of 0.5°/h with an integration time of 1 s. Iwatsuki K et al., 1984, demonstrated improved sensitivity using an RFOG based on a square wave-modulated optical source instead of a He-Ne laser. Xie et al., 2016, developed an active resonator by splicing an erbium-doped fiber into the ring resonator, achieving better sensitivity by improving scale factor stability [60]. For high-performance applications, the optical source was replaced with a narrow linewidth source and phase modulation techniques were introduced by Yao Yi et al., 1995. In order to achieve shot noise-limited sensitivity, the single mode fiber was replaced with a polarization maintaining fiber (PMF) in the ring resonator by Xulin Zhang et al., 2006 and Diqing Ying et al., 2008. In Lishuang Feng et al., 2012 demonstrated an RFOG with a hollow-core photonic bandgap fiber (HC-PBF) to avoid the Kerr effect, and they reported a sensitivity of 0.153°/h. In Shailesh Srivastava et al., 2016 developed an RFOG using a single coupler resonator with reflector (SCRWR) configuration and a 500 kHz linewidth laser source, proposing a potential sensitivity of 0.01°/h. In Sumukh Nandan R et al., 2019 reported a possible sensitivity of less than 1°/h using a novel beat frequency based signal detection method. In Shuang et al., 2022 reported that a broadband source based RFOG with a high frequency modulation demodulation method is able to achieve the theoretical shot noise limited sensitivity.

Although the development of IFOGs and RFOGs started at the same time, the former has been included in inertial measurement units (IMUs) [66,67] while the latter is still in the development stages [68]. This is in spite of their theoretical shot noise-limited sensitivities being the same. An IFOG typically uses a broadband light source at 1550 nm, which is typically based on an erbium-doped fiber amplifier (EDFA) and 1 km-long polarization maintaining fibers (PMFs). RFOGs, on the other hand, use shorter lengths of fibers (a few dozen meters); thus, they are less sensitive to thermal gradient effects. However, conventional RFOGs require highly coherent and extremely narrow linewidth lasers to achieve the same performance as medium-range IFOGs. On the other hand, resonant micro-optic gyroscopes (RMOGs) have been developed with the aim of achieving reasonable performances at reduced sizes.

The bias stability is one of the key limiting factors for most gyroscopes [16]. IFOGs have matched RLGs by reaching a bias stability of <0.001°/h [3]. The bias stabilities achieved during the past 30 years of research and development in RFOGs are listed in Table 3. Strandjord L K et al., 1991, achieved a bias stability of 10°/h using a PMF-based ring resonator with a 90° splice joint. Zhonghe Jin et al., 2012, achieved a bias stability of 9.5°/h using laser frequency stabilization. By using a PMF-based ring resonator and an in-line polarizer, a bias stability of less than 5°/h was demonstrated by Huilian Ma et al., 2012 and Xuhui Yu et al., 2013. An RFOG with a Pound-Drever-Hall (PDH) locking scheme achieved a bias stability of 1°/h (Sanders G A et al., 2017) and 0.45°/h (Ravaille Alexia et al., 2018). By using a photonic crystal fiber (PCF)-based ring resonator and FPGA-based signal processing, a bias stability of 0.5°/h (Yu H et al., 2018) and 0.05°/h (Yong Li et al., 2021) was achieved. A round-trip filtering scheme with a broadband source-based RFOG was recently demonstrated, with a bias stability of 0.012°/h. Additionally, a closed-loop method with a broadband source demonstrated a bias stability of 0.0063°/h [69].

**Figure 5:**
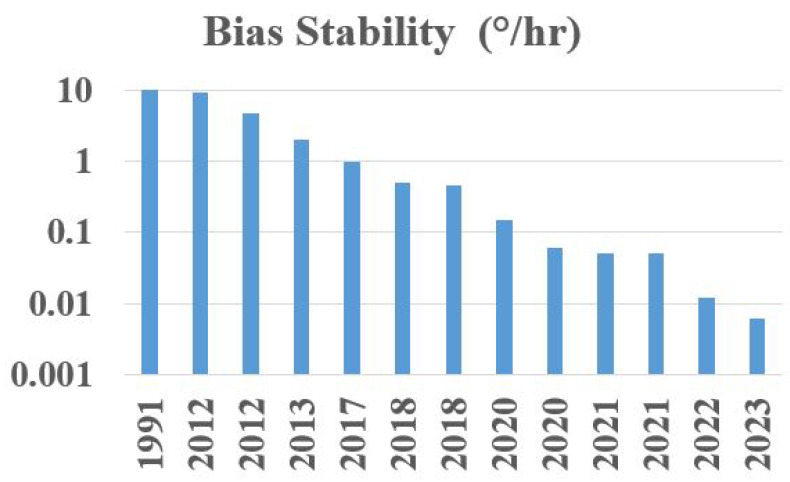
Bar graph showing the improvement in bias stability of RFOGs over the past three decades.

The improvement in bias stability of RFOGs over the past three decades is shown in Figure 5. However, there is a need for further improvement in bias stability for high-performance navigation applications, which require 0.001°/h; there is also a need to reduce complexity and the cost of detection. A few emerging ideas to address these issues are discussed in the following section.

Angular random walk (ARW) is another key parameter that is used to quantify the accuracy of RFOGs. In recent research, the ARW has been improved to meet the requirements for navigation-grade applications, as listed in Table 4. In Strandjord et al., 1992 reported an ARW of 0.1°$/\sqrt{h}$ by using a PM fiber-based ring resonator with 90° polarization rotation. In Strandjord et al., 2012 achieved a navigation-grade ARW of 0.0083°$/\sqrt{h}$ by using an AOM frequency shifter and phase modulation. By using Pound-Drever-Hall (PDH) locking for laser frequency stabilization, Sanders G et al., 2017 demonstrated an ARW of 0.003°$/\sqrt{h}$. In a recent study using a broadband source and a fiber length of 100 m, an ARW value of 0.0093°$/\sqrt{h}$ was achieved. In another recent study, an ARW of 0.0013°$/\sqrt{h}$ was achieved using a fiber length of 200 m, along with high-frequency phase modulation. Note that polarization maintaining fiber (PMF) was used in all the above RFOG experiments to overcome environmentally induced random birefringence in the fiber ring resonator.

## 4. Opportunities for Performance Enhancement in Resonant Fiber Optic Gyroscopes

Several promising avenues to enhance the performance of RFOGs have emerged during the past decade. These are discussed in detail below.

### 4.1. Single-Coupler Resonator with Reflector (SCRWR)

**Figure 6:**
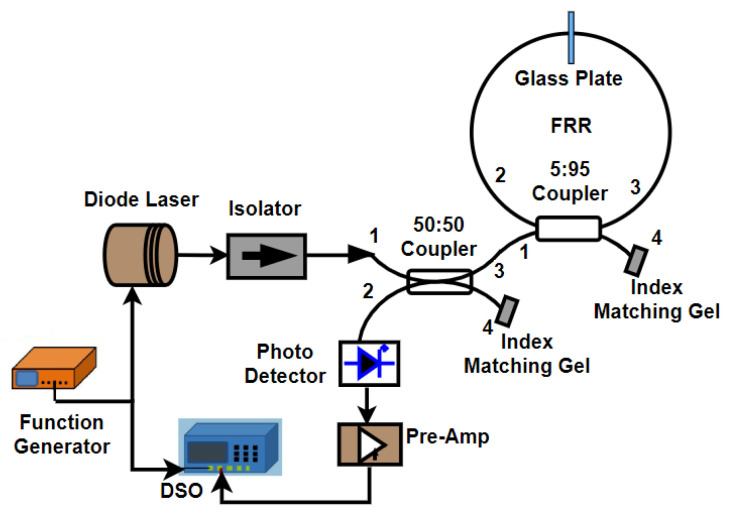
Schematic diagram of a single-coupler resonator with reflector (SCRWR) gyroscope configuration with an intensity-based readout [80].

One of the more promising approaches is a single-coupler resonator with reflector (SCRWR) configuration, as illustrated in Figure 6; an in-line reflector in the resonator deliberately couples the light beam between the two counter-propagating paths. Although this is generally avoided in all the other optical gyroscopes, the advantage of this configuration is that it makes available both the reflectance as well as the transmittance of the resonator for analysis at the same port.

#### 4.1.1. SCRWR: Intensity-Based Readout

The SCRWR configuration was first proposed by Hanna and Paschotta [81] as an application for designing single-frequency lasers using a transfer matrix method. There are several approaches to modeling the workings of optical ring resonators [82,83,84,85]. Novel ring resonators that can offer an enhanced free spectral range due to the Vernier effect have been reported previously [86,87]. A ring resonator with a highly birefringent fiber and an in-line reflector was proposed for creating the narrowband filters required for single-frequency operation [88]. The application of SCRWR in demonstrating single, longitudinal-mode lasing was presented by A Das et al. [89]. A signal flow graph (SFG)-based approach to understanding the transmittance and reflectance properties of SCRWR was first reported by Srivastava et al. [90]. This method, along with Mason’s rule, offers a simpler solution to understanding the coupled cavity nature of the SCRWR method and its application to resonant gyroscopes. The sensitivity is limited by the linewidth of the laser source since the readout scheme in RFOGs is frequency-based. On the contrary, in the SCRWR gyroscope the readout is power-based, and hence, even a finesse of 60 is sufficient, apart from less expensive components, to obtain better sensitivities than those of RFOGs. Moreover, the sensitivity of SCRWR gyroscopes scales linearly with cavity length, unlike RFOGs.

#### 4.1.2. SCRWR: Beat Frequency-Based Readout

**Figure 7:**
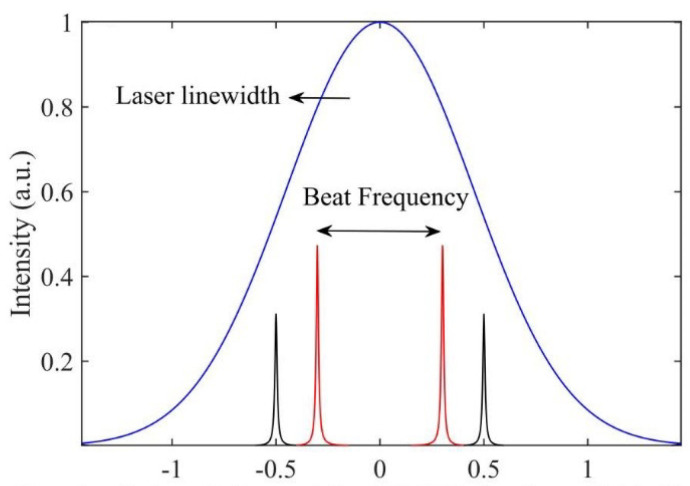
Beat frequency for different rotation induced phase changes [11].

An SCRWR configuration with an in-line reflectance larger than the critical reflectance was developed. In this case, the concept of mode splitting is used to observe the beat frequency corresponding to the angular rate. The reflected spectrum is the spectral filtered output of the input laser spectrum. This filtered spectrum, consisting of two narrow frequencies, generates a beat frequency in the detector output corresponding to the mode splitting, as shown in Figure 7.

The fluctuations in the optical power difference between the CW and CCW beams of the ring resonator can cause the nonreciprocal phase bias of a gyroscope to drift. This creates additional nonreciprocal errors in the bias of the gyroscope, $\Delta \varphi =\Delta \varphi_{b}+\Delta \varphi +\Delta \varphi_{kerr}$. The term $\Delta \varphi_{kerr}$ refers to the difference between the nonlinear phases of the CW and CCW paths. The expression for the Kerr-induced nonlinear phase shift is given in the work of Sumukh Nandan et al. [91]. The powers of the two counter-propagating beams can be made equal by the appropriate choice of the reflectance ‘R’ and coupling coefficient ‘k’ values. For example, using the equations for Kerr nonlinear phase shift [11] for a given phase bias of $\Delta \varphi_{b}$ = 0.058 rad for a loss-compensated case (α=γ=0), R = 0.5%, and k = 11.5%, the values of $P_{cw}$ and $P_{ccw}$ become almost equal at resonance. Thus, the Kerr nonlinearity induced for bias stability can be eliminated via the suitable choice of parameters for a given design.

**Figure 8:**
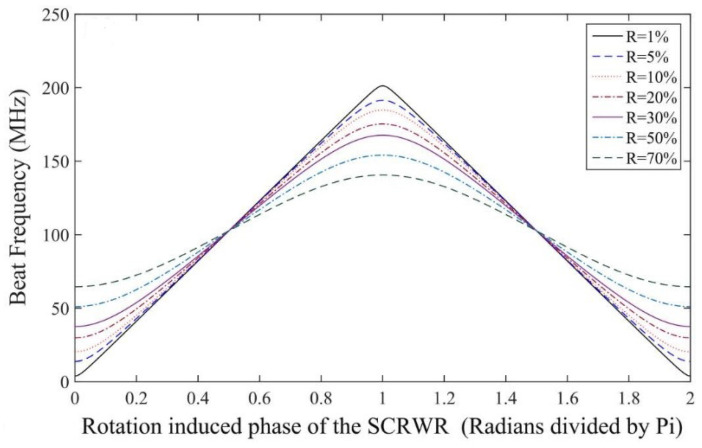
Change in beat frequency vs. rotational bias for different values of reflectivity [11].

Due to the bias value, $\Delta \varphi_{b}$, it is clear from Figure 8 that the beat frequency is around half the FSR for small rotations. The source laser linewidth, therefore, needs to be about half the FSR of the resonator, and this requires a laser of about 100 MHz and 30 MHz for the 1 m and 3 m resonators, respectively. This is a very relaxed requirement compared to those of standard RFOGs, which require extremely narrow linewidth lasers of about 5 to 10 kHz. The cost of lasers with a linewidth in the range of 5 to 100 MHz is generally more than an order of magnitude less than those with linewidths of 5 to 50 kHz. The other factors that can influence sensitivity are the Rayleigh backscattering and thermo-optic effects, such as Shupe effects [92]. Interestingly, as explained previously, the embedded reflector is used to intentionally couple the two counter-propagating beams. Other gyroscope designs avoid this feature. As a result, the weak coupling due to backscattering does not contribute much to the beat frequency measurements. The backscattering from various sections gets summed up with different phases, and this additionally reduces its degrading performance.

### 4.2. Frequency Comb Source-Based RFOG

In a conventional RFOG, a single wavelength laser source is used to generate counter-propagating waves in a ring resonator, for which the difference in resonance frequency is measured to obtain the rotation rate. However, the primary limitation of RFOG performance is bias drift, which is caused by nonreciprocal effects and environmental fluctuations. In order to enhance bias stability, an alternative approach has been investigated based on a frequency comb source (FCS) [93,94]. By using a different set of frequencies (3, 5, 7, 9, etc.) for the counter-propagating waves, the uncertainty in the demodulated phase is diminished compared to a single frequency measurement, leading to enhanced accuracy in the determination of the rotation rate.

**Figure 9:**
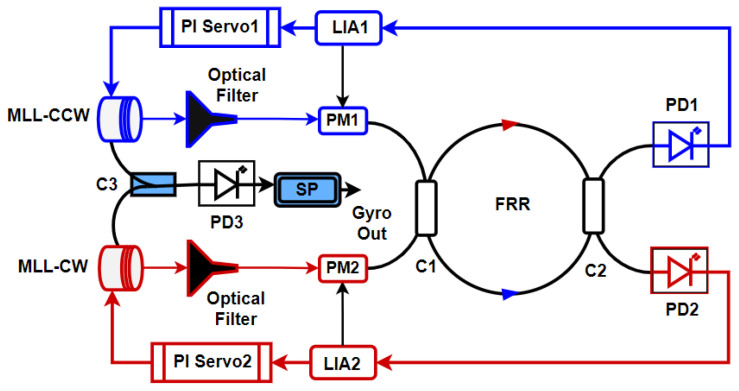
Schematic diagram of a frequency comb source-based RFOG. Two mode-locked lasers are used to generate a separate comb of frequencies for the CW and CCW light beams. The rotation rate is measured by beating the two frequency combs as they are modulated by using FRR resonance shift through closed-loop feedback.

The typical configuration of an RFOG based on a frequency comb source is shown in Figure 9. It consists of two repetition rate tunable mode-locked lasers, each in the CW and CCW directions. The center frequency of both lasers is tuned to the instantaneous resonance frequency of the fiber ring resonator in either direction. However, both the CW and CCW light beams have a frequency comb spectrum in the same spectral range. Since the mode-locked laser repetition rate is 50 MHz, five longitudinal modes for the comb are supported in the ring resonator, for which the free spectral range is 10 MHz. The five fundamental modes are filtered using an optical filter in the CW and CCW directions and phase-modulated using a $LiNbO_{3}$ phase modulator in relation to frequency, $f_{m1}$ and $f_{m2}$, respectively.

**Figure 10:**
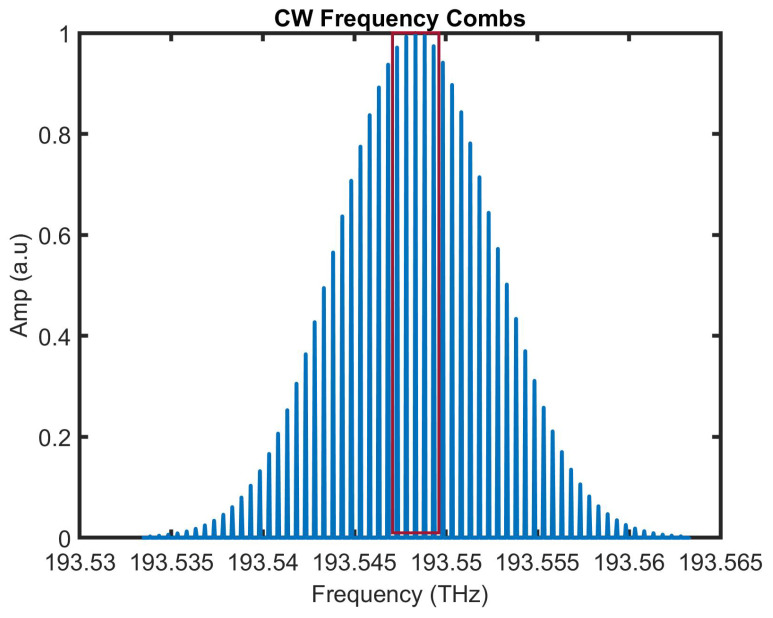
A simulated frequency comb from which a set of frequencies are carved out in the central region.

The simulated frequency comb spectrum for a CW laser is shown in Figure 10. The frequency comb spectrum has a spacing of 50 MHz, and the linewidth for each comb line is approximately 100 kHz. The frequency comb spectrum for a CCW laser source is similar to the CW laser source, with a small shift in the center frequency. The output of the CW and CCW laser sources is filtered using an optical filter to extract five longitudinal modes, as shown red coloured box in Figure 10. Based on the output of the respective lock-in amplifiers, the free spectral range of the CW and CCW mode-locked lasers are adjusted. The beat frequency of the mode-locked lasers is detected, and the signal processing (SP) output gives the rotation rate information. Although such a configuration was proposed almost a decade ago, the complexity of the scheme has hitherto discouraged widespread interest. However, the promise of bias stability enhancement may prompt further research in this area, as compact, highly stable frequency comb sources have seen much development lately.

### 4.3. Hollow-Core Anti-Resonant Fiber-Based RFOG

**Figure 11:**
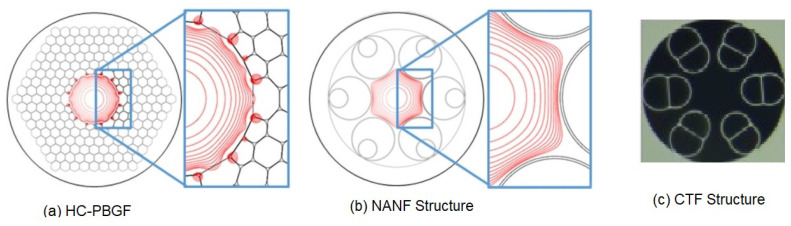
Comparison of (**a**) HC-PBGF, (**b**) NANF structures [75] (**c**), and CTF structures [95].

As discussed earlier, hollow-core fibers are useful in avoiding the nonlinear Kerr effect and thermal drift issues in gyroscopes. However, one of the key challenges to hollow-core fiber (HCF) deployment in an RFOG lies in coherent backscattering. Terrel et al. [52] showed that for HC-PBGF, as in Figure 11a, backscatter severely limits the bias stability of an HCF RFOG [75]. The signal-processing techniques alleviate the primary error mechanisms that arise but are unable to suppress secondary backscatter errors, including those mediated by surface modes in hollow-core photonic bandgap fibers (HC-PBGFs). The use of nested anti-resonant nodeless fiber (NANF), as shown in Figure 11b, considerably improved spatial mode purity and the backscattering of the fiber over photonic bandgap HCFs, which is the key metric of technological advancement in RFOGs. The long-term bias stability was approximately 0.05°/h for observation times of 1–10 h and achieved more than a 100 times longer integration time than prior bias stability demonstrations on HCF-based RFOGs.

A new generation of interferometric air-core FOG based on conjoined-tube anti-resonant fiber (CTF) was demonstrated [95], as shown in Figure 11c. The CTF coil consists of multiple anti-resonant layers, a suitably configured core diameter, sufficient fiber length, low transmission loss, and high modal purity, which are guaranteed, allowing the conjoined-tube anti-resonant fiber optic gyroscope (CTFOG) to achieve a long-term bias stability of 0.016°/h over 1000 s. This also demonstrated (experimentally) a reduction in the thermal sensitivity by a factor of 11.2 in the CTFOG compared to the conventional PMFOG with the same coil length and diameter, which features the air-core FOG with enhanced thermal stability. As these fiber technologies mature further and related devices become more commonly available, we anticipate further exciting developments in this direction.

### 4.4. RFOG Using a Broadband Source

**Figure 12:**
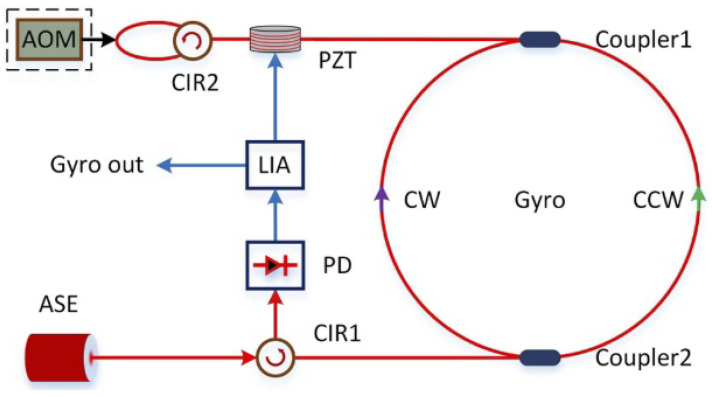
Schematic diagram of an RFOG using an ASE source in a reflected resonator open-loop configuration [76].

In all the RFOG configurations discussed above, we need to use a highly coherent source that needs to be actively locked with respect to a fiber ring resonator (FRR). In order to improve the signal-to-noise ratio at the receiver circuit, a frequency comb source was proposed above instead of the conventional single-line source. However, the requirement of frequency locking the comb source with respect to the longitudinal modes of the FRR through close-loop feedback still remains in this case. The key question, then, is whether it is possible to circumvent the frequency locking requirement. An ingenuous broadband light source-based reflectometer configuration may be the solution. Figure 12 illustrates such a scheme, wherein a portion of the broadband light launched into the FRR is coupled out through Coupler 1 and injected back into the FRR cavity. The injected light helps to cancel any rotation-based frequency shift in the CW direction, thereby providing a highly stable frequency comb source that is naturally locked to the FRR longitudinal modes. Based on this configuration, a bias stability of 0.012°/h was achieved [76]. This is probably the best result achieved for an open-loop gyroscope. The use of a broadband source also helps to mitigate the effects of coherent backscattering and Kerr nonlinearity, which are common issues in narrowband sources, leading to more stable measurements over time. It is remarkable that the best performance achieved for a 1030 m long PM fiber has a bias stability of 0.00045°/h and an angular random walk of 0.00019°$/\sqrt{h}$ [96]. Such a performance from RFOGs provides a viable alternative to mechanical gyroscopes for strategic navigation applications.

## 5. Conclusions

In the last decade, substantial progress has been made to increase the performance of RFOGs for inertial navigation applications. In a conventional RFOG, a single-wavelength laser source is used to generate counter-propagating waves in a ring resonator, for which a phase difference leads to a resonance frequency shift that is measured as an intensity change to obtain the rotation rate. However, the primary limitation of RFOG performance is the bias drift, which can be observed due to nonreciprocal effects, such as Kerr nonlinearity, Rayleigh backscattering, and environmental fluctuations.

In this paper, we reviewed the key challenges of achieving bias stability enhancement in RFOGs. Specifically, the use of a highly coherent source, phase modulation techniques, fiber ring resonator design, and signal-processing methods are discussed. The reported results over the years show that the enhancement in bias stability using a single-wavelength source is reasonably optimized in RFOG.

Even though RFOG performance has already seen rapid improvement in the last few years, we believe that further enhancement is possible. In our last section, we have highlighted several promising new approaches, including the use of reflector configuration, frequency comb sources, hollow-core anti-resonant fibers, and broadband sources. Such approaches are poised to firmly push the bias stability values to less than 0.001°/h, making them highly attractive for navigation applications. 

## Figures and Tables

**Table 1:** Performance parameter comparison of various grades of gyroscopes.

Parameters	Control Grade	Tactical Grade	Inertial Grade
ARW (°$/\sqrt{h}$)	>0.5	0.05–0.5	<0.05
Bias stability (°/h)	>10	0.1–10	<0.1
Scale factor stability (ppm)	>500	100–500	<100

**Table 2:** Sensitivity improvement for various RFOG configurations over the past four decades.

Year	Sensitivity (°/h)	Methods Used	Reference
1983	0.5	SMF and He-Ne laser	[27]
2006	0.144	PMF with phase modulation	[61]
2007	0.0825	PMF with phase modulation	[62]
2012	0.153	HC-PBF based FRR	[63]
2016	0.01	Single coupler resonator with reflector	[64]
2019	<1	Beat-frequency-based detection	[11]
2022	0.001	Broadband source	[65]

**Table 3:** Comparison of bias stabilities for various RFOG configurations over the past three decades.

Year	Bias Stability (°/h)	Methods Used	Reference
1991	10	PMF, 90° splice	[42]
2012	9.5	Frequency stabilization	[70]
2012	4.7	PMF, In-line polarizer	[45]
2013	2	PMF, In-line polarizer	[46]
2017	1	PDH locking scheme	[71]
2018	0.5	PCF, signal processing	[20]
2018	0.45	PDH locking scheme	[55]
2020	0.15	HCPBF with Meniscus lens	[72]
2020	0.06	Reciprocal modulation-demodulation	[73]
2021	0.05	SMF, birefringence	[74]
2021	0.05	Nested anti-resonant nodeless fiber (NANF)	[75]
2022	0.012	Round-trip filtering with a broadband source	[76]
2023	0.0063	Broadband source with closed-loop	[69]

**Table 4:** Angular random walk (ARW) performance for different RFOG configurations.

Year	ARW (°$/\sqrt{h}$)	Methods Used	Reference
1992	0.1	PMF, 90° polarization rotation	[42]
2012	0.0083	AOM frequency shifter with phase modulation	[77]
2017	0.003	PDH locking Scheme	[78]
2022	0.0093	Broadband source, multi-beam interference method	[79]
2022	0.0013	Broadband source, high-frequency phase modulation	[65]

## Data Availability

Data are contained within the article.

## Abbreviations

The following abbreviations are used in this manuscript:

IFOG	Interferometric fiber optic gyroscope
RFOG	Resonant fiber optic gyroscope
MEMS	Micro electro mechanical sensors
RLG	Ring laser gyroscope
RMOG	Resonant micro optic gyroscope
FSR	Free spectral range
FWHM	Full-width half maximum
FPGA	Field programmable gate array
SCRWR	Single-coupler resonator with reflector
IMU	Inertial measurement unit
EDFA	Erbium-doped fiber amplifier
FCS	Frequency comb source
NANF	Anti-resonant nodeless fiber
